# Propyl­amine–borane

**DOI:** 10.1107/S160053680901887X

**Published:** 2009-05-23

**Authors:** Graeme J. Gainsford, Mark E. Bowden

**Affiliations:** a Industrial Research Limited, PO Box 31-310, Lower Hutt, New Zealand

## Abstract

The title compound, C_3_H_12_BN, was solved using data collected from a multiple crystal (note incomplete data shell). The cell packing is dominated by bifurcated attractive N—H^δ+^⋯^δ−^H—B inter­actions.

## Related literature

For background to our studies of hydrogen storage materials and the synthesis: see Bowden *et al.* (2007 ▶, 2008 ▶). For other H_3_B–N-containing boranes, see: Alston *et al.* (1985 ▶); Spielmann *et al.* (2008 ▶). For bond lengths and angles in boranes, see: Ting *et al.* (1972 ▶); Klooster *et al.* (1999 ▶); For hydrogen-bond motifs, see: Bernstein *et al.* (1995 ▶).
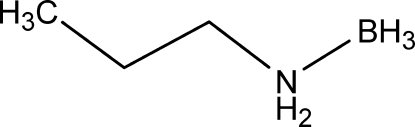


## Experimental

### 

#### Crystal data

C_3_H_12_BN
*M* *_r_* = 72.95
Monoclinic, 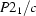

*a* = 9.173 (4) Å
*b* = 8.638 (3) Å
*c* = 7.360 (3) Å
β = 97.892 (8)°
*V* = 577.7 (4) Å^3^
*Z* = 4
Mo *K*α radiation
μ = 0.05 mm^−1^
*T* = 93 K
0.45 × 0.25 × 0.03 mm

#### Data collection

Bruker–Nonius APEXII CCD area-detector diffractometer
Absorption correction: none
846 measured reflections
846 independent reflections
503 reflections with *I* > 2σ(*I*)
*R*_int_ = 0.060

#### Refinement

*R*[*F* ^2^ > 2σ(*F* ^2^)] = 0.051
*wR*(*F* ^2^) = 0.138
*S* = 1.00
846 reflections
57 parameters
H-atom parameters constrained
Δρ_max_ = 0.14 e Å^−3^
Δρ_min_ = −0.14 e Å^−3^

### 

Data collection: *APEX2* (Bruker, 2005 ▶); cell refinement: *SAINT* (Bruker, 2005 ▶); data reduction: *RLATT* (Bruker, 2004 ▶) and *SAINT*; program(s) used to solve structure: *SHELXS97* (Sheldrick, 2008 ▶); program(s) used to refine structure: *SHELXL97* (Sheldrick, 2008 ▶); molecular graphics: *ORTEP-3* (Farrugia, 1997 ▶) and *Mercury* (Macrae *et al.*, 2006 ▶); software used to prepare material for publication: *SHELXL97* and *PLATON* (Spek, 2009 ▶).

## Supplementary Material

Crystal structure: contains datablocks global, I. DOI: 10.1107/S160053680901887X/bt2953sup1.cif

Structure factors: contains datablocks I. DOI: 10.1107/S160053680901887X/bt2953Isup2.hkl

Additional supplementary materials:  crystallographic information; 3D view; checkCIF report

## Figures and Tables

**Table 1:** Hydrogen-bond geometry (Å, °)

*D*—H⋯*A*	*D*—H	H⋯*A*	*D*⋯*A*	*D*—H⋯*A*
N1—H8⋯H11^i^	0.89	2.16	2.96	149
N1—H9⋯H11^ii^	0.89	2.07	2.93	163

Symmetry codes: (i) 
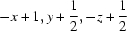
; (ii) 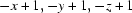.

**Fig. 1.**
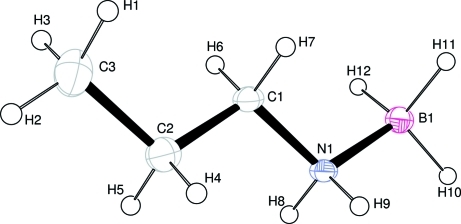
Molecular structure of the asymmetic unit (Farrugia, 1997); displacement ellipsoids are shown at the 30% probability level.

**Fig. 2.**
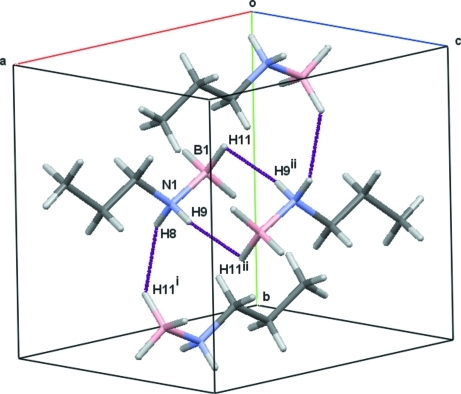
Cell contents view (Mercury; Macrae et al., 2006). For clarity only a limited set of atoms are labelled. Hydrogen bonds are shown as rippled lines (purple) with carbon, nitrogen & boron atoms gray, blue & pink respectively. Symmetry codes: (i) 1 - x, 1/2 + y,1/2 - z (ii) 1 - x,1 - y,1 - z (see Table 2).

## supplementary crystallographic information

### Comment

We have previously reported structures of ammonia borane (Bowden *et al.*, 2007) (FUYVUQ03) and methylamine borane (Bowden *et al.*, 2008) (EFAGEY) as part our studies of hydrogen storage materials. We were challenged to solve the title compound structure by the poorly crystalline platey crystals and to enhance our understanding of the solid state intermolecular interactions. Most other reported H_3_B–N containing boranes are present as solvent in clathrates (*e.g.* DATGAG, Alston *et al.*, 1985); a recent exception is a calcium borylamine complex (VODWUH, Spielmann *et al.*, 2008).

The bond lengths and angles (Fig 1, Table 1) are consistent with those previously reported e.g B–N in solvent ammonia boranes vary from 1.579 to 1.606 Å, whilst the other pure boranes (EDABRO, ethylenediamine-bis(borane) (Ting *et al.*, 1972), FUYVUQ03 & EFAGEY) average 1.592 (12) Å. The non-hydrogen atom chain is effectively coplanar with r.m.s. deviation 0.0136 Å. The refined B–H distance is consistent with previously observed distances of 1.13–1.15 Å.

The molecules are packed utilizing N—H^δ^^+^···^δ^^-^H—B attractive interactions at the N1 hydrogen H11 (Table 2, Figure 2). One links inversion symmetry related molecules (entry 2) resulting in the equivalent of a *R*^2^_2_(8) graphset moiety (Bernstein *et al.*, 1995). The other links screw axis related molecules (entry 1). The H···H distances are similar to those found in EDABRO (2.04,2.12 Å) and by neutron diffraction for ammonia borane (2.02 (3), 2.21 (4), 2.23 (4); Klooster *et al.*, 1999).

### Experimental

Synthesis was carried out using a procedure analogous to that for methylamine borane (Bowden *et al.*, 2008). An equimolar mixture of propylamine hydrochloride (dried at 110°C) and sodium borohydride were stirred in anhydrous tetrahydrofuran in a reaction flask at room temperature. Evolution of hydrogen gas was observed immediately, and overnight 1 mol of hydrogen was collected. The resulting suspension was filtered to remove sodium chloride, and tetrahydrofuran removed by rotary evaporation. A near quantitative yield of propylamine borane crystals remained.

### Refinement

Diffraction data was extracted from the major of multiple intersecting lattices using RLATT (Bruker, 2004). The structure was solved by direct methods but refinement halted at R1 0.18 for the 684 unique data with I>2σ(I). Inspection of data showed a large number with F_o_>>F_c_ indicating coincidental contributions from the other contributing lattice(*s*). A total of 172 reflections which met the two criteria [with q=1.3], (1) I(obs)/I(calc) > q and (2) (I(obs)-I(calc)) > qσ(I(obs)), were then excluded from the dataset. The conventional R1 for these rejected data was 0.44. The ratio criteria q was varied down to values of 0.9: although the R1 agreement factors converged at around a ratio of 1.0 (R1 0.051, for 491 I>2σ(I) data) no significant changes occurred in final su values or parameters compared with the slightly larger dataset. On the basis that another analysis of the data would be possible if the larger dataset was presented, the refinement was continued with the (ratio 1.3) 512 independent remaining reflections (R1 0.0544). Nine further weak intensity reflections at high theta, outliers with I(obs)>>I(calc), were then omitted lowering R1 to 0.0508 for the final dataset (503 I>2σ(I)).

The *X*—H bond distances (where *X* = C1, C2, B1 & N1) were refined. All methyl and other H atoms were refined as riding on their parent atom with *U*_iso_ 1.5 & 1.2 times respectively that of the *U*_eq_ of their parent atom.

### Crystal data

**Table:**

C_3_H_12_BN	*F*(000) = 168
*M**_r_* = 72.95	*D*_x_ = 0.839 Mg m^−^^3^
Monoclinic, *P*2_1_/*c*	Mo *K*α radiation, λ = 0.71073 Å
Hall symbol: -P 2ybc	Cell parameters from 1112 reflections
*a* = 9.173 (4) Å	θ = 3.3–26.4°
*b* = 8.638 (3) Å	µ = 0.05 mm^−^^1^
*c* = 7.360 (3) Å	*T* = 93 K
β = 97.892 (8)°	Plate, colourless
*V* = 577.7 (4) Å^3^	0.45 × 0.25 × 0.03 mm
*Z* = 4	

### Data collection

**Table:**

Bruker–Nonius APEXII CCD area-detector diffractometer	503 reflections with *I* > 2σ(*I*)
Radiation source: fine-focus sealed tube	*R*_int_ = 0.060
graphite	θ_max_ = 25.1°, θ_min_ = 3.7°
Detector resolution: 8.192 pixels mm^-1^	*h* = −10→10
φ and ω scans	*k* = 0→10
846 measured reflections	*l* = 0→8
846 independent reflections	

### Refinement

**Table:**

Refinement on *F*^2^	Primary atom site location: structure-invariant direct methods
Least-squares matrix: full	Secondary atom site location: difference Fourier map
*R*[*F*^2^ > 2σ(*F*^2^)] = 0.051	Hydrogen site location: inferred from neighbouring sites
*wR*(*F*^2^) = 0.138	H-atom parameters constrained
*S* = 1.00	*w* = 1/[σ^2^(*F*_o_^2^) + (0.0567*P*)^2^ + 0.1469*P*] where *P* = (*F*_o_^2^ + 2*F*_c_^2^)/3
846 reflections	(Δ/σ)_max_ < 0.001
57 parameters	Δρ_max_ = 0.14 e Å^−^^3^
0 restraints	Δρ_min_ = −0.14 e Å^−^^3^

### Special details

**Table:**

Geometry. All e.s.d.'s (except the e.s.d. in the dihedral angle between two l.s. planes) are estimated using the full covariance matrix. The cell e.s.d.'s are taken into account individually in the estimation of e.s.d.'s in distances, angles and torsion angles; correlations between e.s.d.'s in cell parameters are only used when they are defined by crystal symmetry. An approximate (isotropic) treatment of cell e.s.d.'s is used for estimating e.s.d.'s involving l.s. planes.
Refinement. Refinement of *F*^2^ against ALL reflections. The weighted *R*-factor *wR* and goodness of fit *S* are based on *F*^2^, conventional *R*-factors *R* are based on *F*, with *F* set to zero for negative *F*^2^. The threshold expression of *F*^2^ > σ(*F*^2^) is used only for calculating *R*-factors(gt) *etc*. and is not relevant to the choice of reflections for refinement. *R*-factors based on *F*^2^ are statistically about twice as large as those based on *F*, and *R*- factors based on ALL data will be even larger.

### Fractional atomic coordinates and isotropic or equivalent isotropic displacement parameters (Å^2^)

**Table:**

	*x*	*y*	*z*	*U*_iso_*/*U*_eq_	
N1	0.57394 (18)	0.52001 (19)	0.2744 (2)	0.0238 (5)	
H8	0.5692	0.5971	0.1940	0.029*	
H9	0.5954	0.5610	0.386	0.029*	
C1	0.6964 (2)	0.4155 (2)	0.2415 (3)	0.0257 (6)	
H6	0.6737	0.3705	0.1238	0.031*	
H7	0.7038	0.3345	0.3288	0.031*	
C2	0.8429 (2)	0.4958 (3)	0.2528 (3)	0.0322 (7)	
H4	0.8675	0.5373	0.370	0.039*	
H5	0.8353	0.5779	0.1690	0.039*	
C3	0.9647 (3)	0.3874 (3)	0.2123 (4)	0.0451 (8)	
H1	0.9727	0.3009	0.2992	0.068*	
H2	1.0582	0.4439	0.2245	0.068*	
H3	0.9418	0.3475	0.0870	0.068*	
B1	0.4156 (3)	0.4415 (3)	0.2603 (3)	0.0270 (6)	
H10	0.3330	0.5297	0.2937	0.038 (4)*	
H11	0.4196	0.3425	0.3603	0.038 (4)*	
H12	0.3825	0.3969	0.1169	0.038 (4)*	

### Atomic displacement parameters (Å^2^)

**Table:**

	*U*^11^	*U*^22^	*U*^33^	*U*^12^	*U*^13^	*U*^23^
N1	0.0366 (11)	0.0151 (9)	0.0210 (10)	−0.0006 (7)	0.0080 (8)	−0.0003 (7)
C1	0.0373 (13)	0.0181 (11)	0.0223 (12)	0.0025 (9)	0.0066 (9)	0.0006 (8)
C2	0.0389 (15)	0.0311 (13)	0.0268 (13)	0.0016 (11)	0.0053 (10)	0.0013 (10)
C3	0.0395 (16)	0.0542 (17)	0.0432 (16)	0.0078 (13)	0.0109 (12)	0.0059 (12)
B1	0.0372 (15)	0.0226 (13)	0.0216 (13)	−0.0017 (11)	0.0056 (10)	−0.0002 (9)

### Geometric parameters (Å, °)

**Table:**

N1—C1	1.487 (3)	C2—H4	0.9359
N1—B1	1.593 (3)	C2—H5	0.9359
N1—H8	0.8880	C3—H1	0.9800
N1—H9	0.8880	C3—H2	0.9800
C1—C2	1.504 (3)	C3—H3	0.9800
C1—H6	0.9465	B1—H10	1.1255
C1—H7	0.9465	B1—H11	1.1255
C2—C3	1.518 (3)	B1—H12	1.1255
			
C1—N1—B1	115.68 (17)	C1—C2—H5	109.1
C1—N1—H8	108.4	C3—C2—H5	109.1
B1—N1—H8	108.4	H4—C2—H5	107.9
C1—N1—H9	108.4	C2—C3—H1	109.5
B1—N1—H9	108.4	C2—C3—H2	109.5
H8—N1—H9	107.4	H1—C3—H2	109.5
N1—C1—C2	113.60 (17)	C2—C3—H3	109.5
N1—C1—H6	108.8	H1—C3—H3	109.5
C2—C1—H6	108.8	H2—C3—H3	109.5
N1—C1—H7	108.8	N1—B1—H10	109.5
C2—C1—H7	108.8	N1—B1—H11	109.5
H6—C1—H7	107.7	H10—B1—H11	109.5
C1—C2—C3	112.4 (2)	N1—B1—H12	109.5
C1—C2—H4	109.1	H10—B1—H12	109.5
C3—C2—H4	109.1	H11—B1—H12	109.5

### Hydrogen-bond geometry (Å, °)

**Table:**

*D*—H···*A*	*D*—H	H···*A*	*D*···*A*	*D*—H···*A*
N1—H8···H11^i^	0.89	2.16	2.96	149
N1—H9···H11^ii^	0.89	2.07	2.93	163

Symmetry codes: (i) −*x*+1, *y*+1/2, −*z*+1/2; (ii) −*x*+1, −*y*+1, −*z*+1.
